# A review of caracal and jungle cat diets across their geographical ranges during 1842–2021

**DOI:** 10.1002/ece3.10130

**Published:** 2023-05-25

**Authors:** Jamshid Parchizadeh, Sarah L. Schooler, Mohammad Ali Adibi, Mariano G. Arias, Sahar Rezaei, Jerrold L. Belant

**Affiliations:** ^1^ Department of Fisheries and Wildlife Michigan State University East Lansing Michigan USA; ^2^ Global Wildlife Conservation Center State University of New York College of Environmental Science and Forestry Syracuse New York USA; ^3^ Department of Habitats and Biodiversity, Faculty of Environment and Energy Islamic Azad University Tehran Iran; ^4^ Environmental Biology Department State University of New York College of Environmental Science and Forestry Syracuse New York USA; ^5^ Department of Biological Sciences, Faculty of Science Engineering University of Arkansas Little Rock Arkansas USA

**Keywords:** caracal, *Caracal caracal*, diet, *Felis chaus*, jungle cat

## Abstract

Co‐occurring carnivore species that are phylogenetically related or of similar size, morphology, and ecological needs often reduce competition by partitioning shared resources through temporal, spatial, and dietary niche segregation via behavioral adaptations. Caracals (*Caracal caracal*) and jungle cats (*Felis chaus*) co‐occur in portions of their geographical ranges and are expected to display resource segregation in these ranges. We compiled scat, stomach content, and prey remains found data from published and unpublished sources to summarize information on the diets of caracals and jungle cats across their geographical ranges during 1842–2021. We obtained 63 sources from 26 countries in Europe, Asia, and Africa, in which caracal diet included 151 species while jungle cat diet included 61 species. We found that caracals and jungle cats did not exhibit dietary niche partitioning and had greater dietary similarities in areas of range overlap. We also found that caracals consumed more diverse prey species including prey with greater average body mass compared to jungle cats. Our results suggest that greater prey diversity in areas of range overlap, caracal predation on wide range of prey, and opportunistic feeding behavior that facilitates consumption of more diverse prey species compared to jungle cats, may facilitate co‐occurrence between these two felid species.

## Graphical abstract

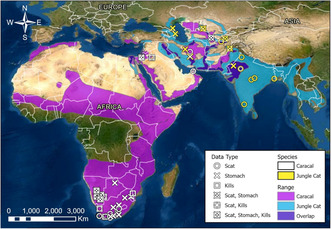

## 1 INTRODUCTION

Diet largely defines the ecological niche of individuals and species (Lanszki et al., 2020). Understanding the diet of carnivores can inform us of their role in ecosystems (Ćirović et al., 2016; Ripple et al., 2014; Roemer et al., 2009), potential effects on prey populations (Głowaciński & Profus, 1997; Klare et al., 2010) including livestock (Banerjee et al., 2013; Kamler et al., 2012), and potential competition with co‐occurring species (Azevedo et al., 2006; Fedriani et al., 1999; Loveridge & Macdonald, 2003). Co‐occurring carnivores that are phylogenetically related or of similar size, morphology, and ecological needs often reduce the negative effects of interactions by partitioning shared resources (Donadio & Buskirk, 2006; Mwampeta et al., 2020; Vanak et al., 2013). Resource partitioning between co‐occurring species can occur through temporal, spatial, and dietary niche segregation via behavioral adaptations (Bianchi et al., 2016; Du Preez et al., 2017; Mwampeta et al., 2020; Wereszczuk & Zalewski, 2015). Consequently, dietary niche partitioning has facilitated the co‐occurrence of numerous carnivore species (Noor et al., 2017; Vanak et al., 2013).

Since the 1990s, mammalian carnivores have emerged as a paradigmatic group of model species to understand the behavioral mechanisms facilitating co‐occurrence, mainly focused on niche theory and dietary niche segregation (Di Bitetti et al., 2010; Glen & Dickman, 2008; López‐Bao et al., 2016). For example, differential prey selection can facilitate co‐occurrence between lion (*Panthera leo*) and leopard (*Panthera pardus*) by reducing competition (Du Preez et al., 2017). Furthermore, larger‐bodied predators overall kill larger‐bodied prey (Carbone et al., 1999) and more diverse prey than smaller‐bodied predators (Cohen et al., 1993), which can facilitate dietary niche segregation. In Sariska Tiger Reserve, India, leopards that had largely fed on rodents before the tiger (*Panthera tigris*) population decline switched to large herbivores after the population decline (Mondal et al., 2011).

The caracal (*Caracal caracal*) is a medium‐sized felid (average body mass = 17 kg; Inskip & Zimmermann, 2009) with a geographic range across Africa and Asia including the Middle East, that can feed on prey nearly twice its size (Avgan et al., 2016; Kohn et al., 2011; Marker & Dickman, 2005; Moqanaki et al., 2016; Nowell & Jackson, 1996) but are also opportunistic and consume diverse vertebrate prey (Drouilly et al., 2020; Farhadinia et al., 2007; Moqanaki et al., 2016; Ünal et al., 2020). Caracals also scavenge larger‐bodied prey species including springbok (*Antidorcas marsupialis*; Avenant & Nel, 2002; Palmer & Fairall, 1988) and bontebok (*Damaliscus pygargus*; Leighton et al., 2020) in South Africa. In contrast, jungle cats (*Felis chaus*, average body mass = 10 kg; Inskip & Zimmermann, 2009) range from south‐eastern Asia to the Middle East and the Caucasus region including Georgia and southern Russia (Chatterjee et al., 2020; Majumder et al., 2011) and consume less diverse prey species (Baker et al., 2003) compared to caracals.

The ecology of free‐ranging species can be best understood through their diet (Litvaitis, 2000), and investigations of felids with similar dietary components are a means for understanding how closely related species use food resources in potentially competitive situations (e.g., where their ranges overlap; Silva‐Pereira et al., 2011). Our objective was to better characterize the diets of these two carnivores using published and unpublished information. We hypothesized that these species would exhibit niche partitioning through dietary separation. Specifically, we predicted that caracals and jungle cats would have greater dietary similarities (in terms of prey categories) in areas without range overlap, and lower dietary similarities in areas of range overlap. We also expected that due to caracals' larger size, they would consume more diverse prey and also consume prey with greater average body mass compared to jungle cats.

## 2 MATERIALS AND METHODS

### 2.1 Literature search and compilation

We used Web of Science (Clarivate) and Google Scholar using the key words “caracal,” “*Caracal caracal*”, “jungle cat”, and “*Felis chaus*”. We examined titles and abstracts of search results for sources that included information on caracal or jungle cat diet. We used bibliographies of Web of Science and sources in Google Scholar to examine sources that cited previously found sources to find further relevant sources. We repeated this process until we did not find new sources.

We compiled a list of species, genera, families, orders, and classes of prey consumed by caracals and jungle cats using the Global Bio Information Database Backbone Taxonomy (GBIF Secretariat, 2021) for prey taxonomy. When possible, we attempted to find original data sources but for some republished data or prey observations, the cited source was unavailable in which case we recorded the citing source and used the republished dataset, therefore our term “data sources” includes republished data and prey observations. We compiled all scat and stomach contents from data sources. We also compiled prey remains found data to determine the total prey base of caracal. For studies where data were collected across multiple study areas, we considered each a separate dataset. For studies or sources that published the same dataset, we used the most complete dataset available from the original author. We recorded sources publishing duplicated data in our species list if they provided additional information to the primary data.

We created categories of prey species by grouping scat, stomach, and prey remains found data based on taxonomic orders for mammals and classes for non‐mammals. Mammal prey species were categorized as small mammals (i.e., Rodentia, Euliptophyla, Macroscelidea), lagomorphs, artiodactyls, livestock, hyraxes, carnivores, and other mammals (i.e., Primates, Tubulidentata, Chiroptera, unknown mammals). Birds, reptiles, amphibians, fish, and arthropods were also represented. When data were published using lower‐rank taxonomy, we summarized data to class‐order categories. We grouped amphibians and reptiles for scat analysis as they were combined in some studies (Heptner & Sludskii, 1992; Khan & Beg, 1986).

### 2.2 Iran stomach contents data

Caracals and jungle cats killed by vehicle collisions, poaching, and herding dogs were collected by us during April 2012–February 2021 (Table S1) and used opportunistically for this study. The hair of prey passes undigested through the predator's gut (Karanth & Sunquist, 1995; Mukherjee et al., 1994). We compared features of hair (e.g., general appearance, color, relative length and width) with references of Iran's Department of the Environment (e.g., Etemad, 1978, 1985; Firouz, 1983, 2012; Harrington & Dareshuri, 1977; Rabiei, 2003; Ziaie, 1996, 2008) to identify prey species.

### 2.3 Analysis

We converted scat data when necessary from number of scats containing a prey item (“frequency of occurrence in scat”) to relative frequency (number of prey items in a category/total number of prey items; Drouilly et al., 2018; Lanszki et al., 2006) by assuming one prey item per prey species per scat. This conversion could result in underestimates of frequencies, particularly for smaller prey species, as there might be more than one individual present in a single scat (Mukherjee et al., 2004). Some of the data were provided only in relative frequency of prey, without numerical prey item observations, necessitating use of relative frequency. We converted seven studies on caracal and four on jungle cat (all scat data) from frequency of occurrence to relative frequency by first summing the total number of prey items in scats, then dividing the number of prey items in each category by total number of prey items. Many studies, even those that reported relative frequency, did not record the number of individuals per species per scat. If relative frequency data included arthropods or plants, we multiplied relative frequency by the total number of prey items found to obtain number of prey items in each category, then removed the arthropod or plant items from the total, and recalculated relative frequency. We excluded arthropods from further analyses because few studies included arthropods in their datasets.

Stomach data was primarily presented as relative frequency or number of prey items consumed, and many stomachs included more than one prey item per species (Heptner & Sludskii, 1992). We omitted two datasets from analysis that presented frequency of occurrence in stomachs (Heptner & Sludskii, 1992; Pringle & Pringle, 1979), to avoid potential errors derived from conversion of frequency of occurrence to relative occurrence. We converted stomach data presented as number of prey items per category to relative frequency. We converted prey remains found data from number of prey identified at prey remains found in a given category to relative frequency of prey at prey remains found. We did not analyze data from one caracal study and two jungle cat studies because data were combined from scat, stomach contents, and/or prey remains found.

To classify studies where the geographical ranges of caracals and jungle cats overlapped, we used range maps from the International Union for the Conservation of Nature (IUCN) Red List (IUCN, 2016, 2020) for sources published since 1990 and historical range maps (Heptner & Sludskii, 1992; Pacifici et al., 2019) for sources published before 1990. When possible, we used locations of studies from coordinates or study location maps from the data source. When coordinates or maps were not provided, we used the best available descriptors to estimate locations. When a study area was described as a region rather than coordinates, we used the center of the study area if coordinates were provided, and the visual center of the described or mapped study area if coordinates were not provided.

Using study locations, we identified datasets derived from areas where the range of caracal and jungle cat overlapped or did not. We further categorized study locations according to dry or wet habitat using the WorldClim 2.1 mean annual precipitation dataset (Fick & Hijmans, 2017) in ArcGIS Pro 3.0.3 (ESRI). This dataset provides downscaled estimates of climate variables (i.e., 12 monthly and one annual mean precipitation layers) based on interpolated station measurements. We used mean annual precipitation of 400 mm as the cutoff for dry and wet habitats (i.e., mean annual precipitation <400 mm as “dry”, and ≥400 mm as “wet”; Hamer & Herrero, 1987; Seymour et al., 2015). We then calculated the sample‐weighted average and standard error of relative frequencies of categorized prey of caracals and jungle cats for all datasets (Gatz & Smith, 1995; Harrell, 2021). Using the sample‐weighted averages, we used two‐way *χ* ^2^ tests to compare caracal and jungle cat prey item composition in scat and stomach contents for both species in areas of range overlap and without overlap. We also calculated the Gini‐Simpson diversity index (1 − *λ*; Hill, 1973) using number of prey items in each category for each dataset (scat overall, with overlap, without overlap, and stomach contents). We tested differences in caracal and jungle cat prey in areas of range overlap and without overlap with scat data only because most sources that provided stomach contents data did not contain specific location information.

To determine caracal and jungle cat dietary similarities, we used the weighted means of categorical prey composition from all four datasets to calculate the Pianka index of niche overlap (Pianka *O*; Pianka, 1973) and Schoener's measure of niche equivalency (Schoener's *D*; Schoener, 1970). The Pianka *O* and Schoener's *D* estimate degree of niche overlap on a scale of 0–1, with a value closer to one suggesting higher niche overlap. We calculated means and 95% confidence intervals of 1000 bootstrapped calculations of the two indices using the “spaa” package in R (Gotelli, 2000; R Core Team, 2021; Zhang, 2016). We bootstrapped 95% confidence intervals to compare dietary similarities of caracals and jungle cats based on scat in areas of range overlap and without overlap. To identify potential influences of habitat type (dry vs. wet) and range overlap (with and without overlap) on caracal and jungle cat dietary similarity by prey weight, we used multiple linear regression using the function *lm* in the R “stats” package (R Core Team, 2021), controlling for multicollinearity with the *vif* function of the R “car” package (Fox & Weisberg, 2019). We treated prey average body weight as our response variable, and predator species, habitat type, and range overlap as our explanatory variables in a three‐way interaction model. We further compared marginal means in a post hoc pairwise comparisons with a Tukey HSD test (Nanda et al., 2021).

## 3 RESULTS

**FIGURE 1:**
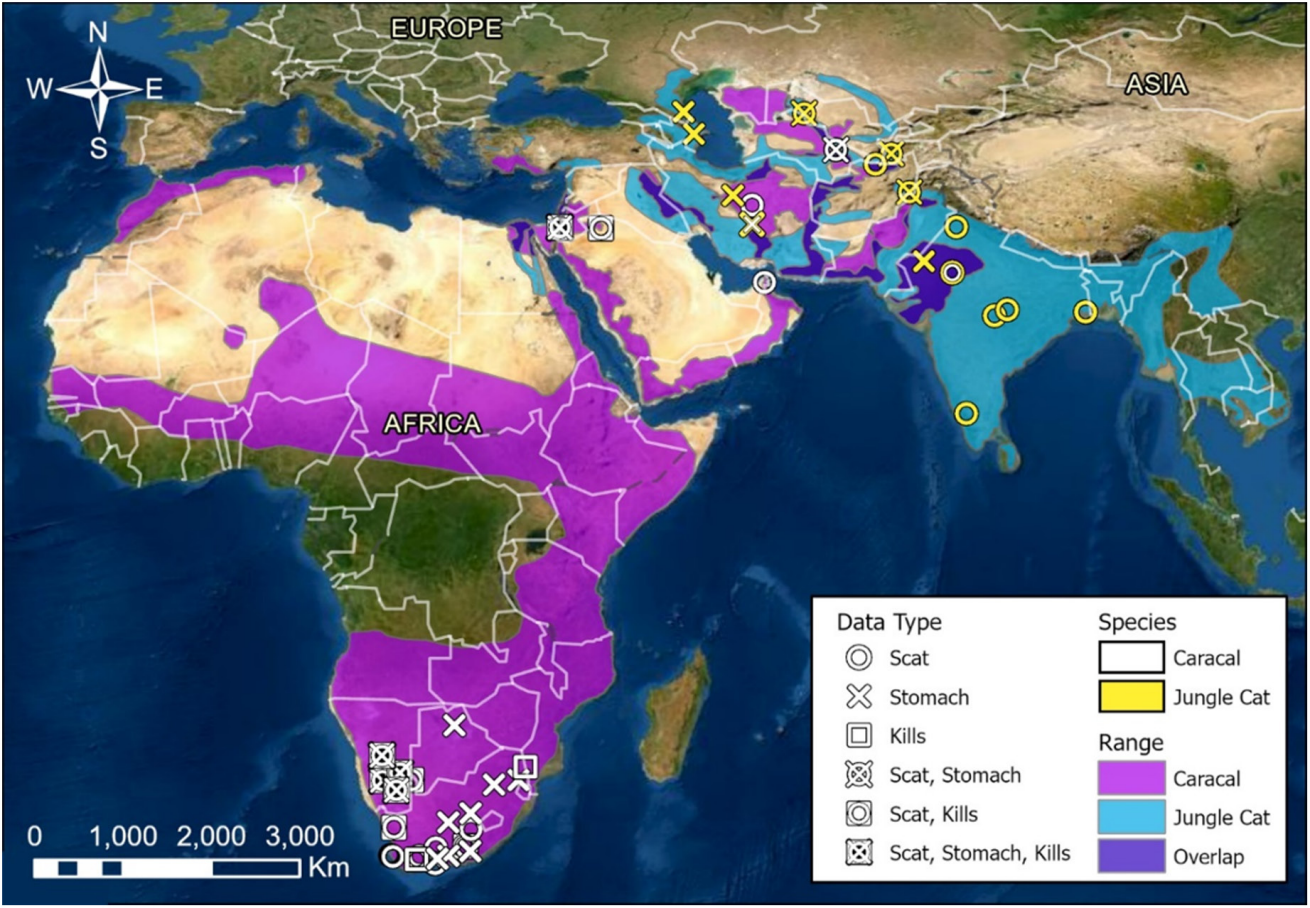
Locations of studies used for caracal (*Caracal caracal*) and jungle cat (*Felis chaus*) diet comparison during 1842–2021 with International Union for the Conservation of Nature (IUCN) species ranges (IUCN, 2016, 2020) with areas of range overlap. For datasets where specific location information was not provided, the location is identified as the center of the country where the study occurred.

We found 63 sources published during 1842–2021 from 26 countries in Europe, Asia, and Africa, five of which provided republished species observations or diet data. We obtained prey species, scat, and stomach contents and prey remains found data from 81 separate sources which were used to determine the total prey base of caracal and jungle cat (Table 1; Figure 1; Appendix S1). Caracal diet included 151 species while jungle cat diet included 61 species (Table 2; Appendices S2 and S3). Both species consumed mammals, birds, amphibians, reptiles, arachnids, and insects, but only caracals consumed millipedes (Diplopoda) and only jungle cats consumed fish (Ichthys; Table 2; Appendices [Link], [Link], [Link]).

**TABLE 1:** Number of data sources for scat; stomach contents (stomach); prey remains found (kills); combined scat, stomach contents, and/or prey remains found data (combined); first or secondhand reports (reports); unverified citations (citations); and author statements without citations (secondary) for caracal (*Caracal caracal*) and jungle cat (*Felis chaus*) diet from 63 data sources (43 caracal, 10 jungle cat, 10 both), 1842–2021.

Species	Scat	Stomach	Kills	Combined	Reports	Citations	Secondary
Caracal	18	9	12	4^a^	10	7	5
Jungle cat	6	4	0	0	2	1	0
Both	1	2	0	0	5	0	3

*Note*: Data sources that provided multiple types of data are in multiple columns.

^a^ Neils (2018) reported data for scat, stomach contents, and prey remains found grouped by category, but only provided a species list for combined data, therefore is placed in all four relevant columns.

**TABLE 2:** Classification of species reported as consumed by caracal (*Caracal caracal*) and jungle cat (*Felis chaus*) from 63 data sources (43 caracal, 10 jungle cat, 10 both), 1842–2021.

	Class	Species	Genera	Families	Orders
Caracal	Mammalia	72	63	29	9
	Aves	65	56	30	19
	Amphibia	1	1	1	1
	Reptilia	13	15	13	1
	Arachnida	0	0	0	1
	Diplopoda	0	0	0	1
	Insecta	0	0	0	4
Jungle cat	Mammalia	27	26	13	6
	Aves	27	28	13	10
	Amphibia	1	1	1	1
	Reptilia	5	9	7	3
	Ichthys	1	2	2	2
	Arachnida	0	0	0	2
	Insecta	0	1	1	1

**FIGURE 2:**
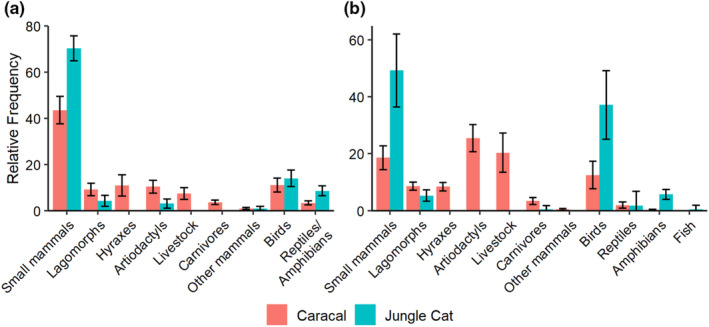
Mean relative frequency weighted by study sample size of prey categories in scat (a) and stomach contents (b) of caracal (*Caracal caracal*) and jungle cat (*Felis chaus*) with 95% weighted standard errors from data collected during 1842–2021.

We used 32 scat datasets for analysis of caracal (*n* = 25) and jungle cat (*n* = 7) diets (Table 3). Categorical distribution of prey items in caracal and jungle cat scat differed (*χ* ^2^ = 440.39, df = 8, *n* = 5513, *p* < .001), with caracal scats containing proportionally more lagomorphs, artiodactyls, hyraxes, livestock, and carnivores, while jungle cat scats contained proportionally more small mammals, birds, and reptiles/amphibians (Figure 2a).

**TABLE 3:** Number of studies (*N*) and the Gini‐Simpson index of diversity (1 − *λ*) for caracal (*Caracal caracal*) and jungle cat (*Felis chaus*) scat and stomach contents (stomach) data in areas of range overlap and without overlap, 1842–2021.

	Caracal		Jungle cat		Pianka *O*		Schoener's *D*	
	*N*	1 − *λ*	*N*	1 − *λ*	*X*	95% CI	*X*	95% CI
Scat	25	0.76	7	0.54	0.94	0.45, 0.99	0.76	0.37, 0.91
Areas of range overlap	2	0.65	3	0.55	0.96	0.71, 1	0.85	0.58, 0.99
Areas without range overlap	23	0.75	4	0.51	0.94	0.53, 0.99	0.78	0.38, 0.91
Stomach	9	0.83	6	0.99	0.55	0.08, 0.94	0.39	0.09, 0.75

*Note*: Also included are means (*X*) and 95% confidence intervals (CI) for the Pianka index of niche overlap (Pianka *O*) and Schoener's measure of niche equivalency (Schoener's *D*) calculated by bootstrapping 1000 iterations.

We used 15 stomach contents datasets for diet analysis of caracal (*n* = 9) and jungle cat (*n* = 6) diets (Table 3), which included the stomach contents data from Iran. Sample size for Iran was 56 stomachs for jungle cats and 31 stomachs for caracals. Categorical distribution of prey items in caracal and jungle cat stomach contents differed (*χ* ^2^ = 216.61, df = 10, *n* = 1064, *p* < .001), with caracal stomach contents containing proportionally more lagomorphs, artiodactyls, livestock, hyraxes, and carnivores, while jungle cat stomach contents contained proportionally more small mammals, birds, and amphibians/reptiles (Figure 2b).

**FIGURE 3:**
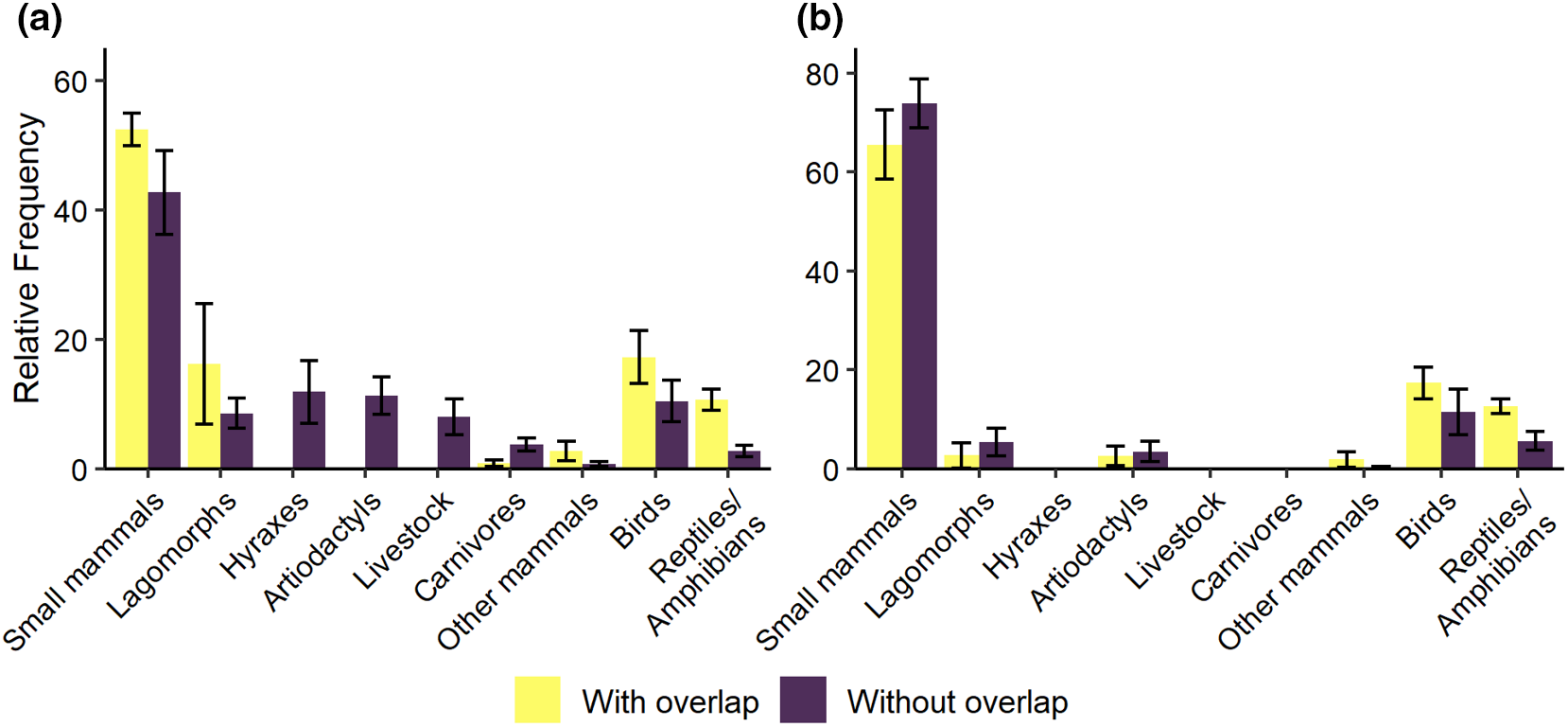
Mean relative frequency weighted by study sample size for prey categories in scat with weighted standard errors for caracal (*Caracal caracal*; a) and jungle cat (*Felis chaus*; b) in areas of range overlap and without range overlap from data collected during 1842–2021.

Categorical distribution of prey items in scats differed between areas of range overlap and without range overlap for caracal (*χ* ^2^ = 154.59, df = 8, *n* = 4321, *p* < .001), and jungle cat (*χ* ^2^ = 20.14, df = 5, *n* = 1063, *p* = .001). Caracal scats in areas of range overlap contained more small mammals, lagomorphs, birds, and reptiles/amphibians, while areas without range overlap contained proportionally more hyraxes, artiodactyls, livestock, and carnivores (Figure 3a). Jungle cat scats in areas of range overlap contained proportionally more birds and reptiles/amphibians, while scat contained proportionally more small mammals in areas without range overlap (Figure 3b).

Prey species in caracal and jungle cat scats were less diverse in areas of range overlap than areas without range overlap (Table 3). Caracal and jungle cat weighted average categorical relative frequency of prey in scats suggested high overlap based on Pianka *O* and Schoener's *D*. There were slightly greater dietary similarities between caracals and jungle cats in areas of range overlap (Pianka *O* = 0.96, Schoener's *D* = 0.85) than in areas without range overlap (Pianka *O* = 0.94, Schoener's *D* = 0.78). Caracal consumed prey with greater average body mass than the jungle cat (*p* ˂ .05) in both areas with and without range overlap, regardless of habitat.

Prey in jungle cat stomach contents was more diverse than prey in caracal stomach contents (Table 3). Caracal and jungle cat weighted average relative frequency of prey in each category in stomach contents suggested low to moderate overlap based on Pianka *O* and Schoener's *D*.

## 4 DISCUSSION

Caracals and jungle cats did not exhibit dietary niche partitioning and had greater dietary similarities in areas of range overlap, contrary to our prediction. However, as predicted caracals did consume more diverse prey species including mammals with greater average body mass compared to jungle cats.

Observed greater dietary similarities between caracals and jungle cats in areas of range overlap could be a consequence of more diverse prey in these areas. Co‐occurring oncillas (*Leopardus tigrinus*), ocelots (*L. pardalis*), and margays (*L. wiedii*) exhibited greater dietary similarities in the Atlantic Rainforest, Brazil, due to more diverse resources (Wang, 2002). Similarly, diets of co‐occurring red foxes (*Vulpes vulpes*) and European badgers (*Meles meles*) converged when resources were more diverse but diverged when resources were less diverse (Barrull et al., 2014; Prigioni et al., 2008; Torretta et al., 2016). Furthermore, greater dietary similarities could be a consequence of temporal or spatial segregation, rather than only dietary niche segregation. Alternatively, though prey abundance was not assessed in our study, we suspect that greater dietary similarities could be associated with greater abundance of small mammals (e.g., rodents) in areas of range overlap as these were the dominant prey of caracals and jungle cats. Co‐occurring predators often consume the most abundant prey species. Rodents were the major diet component of co‐occurring dingoes (*Canis lupus dingo*) and red foxes during a rodent population outbreak (Pavey et al., 2008), and oncillas, jaguarundis (*Puma yagouaroundi*), and ocelots co‐occurred without competing for food (Silva‐Pereira et al., 2011) because rodents were abundant prey (Solari & Rodrigues, 1997). Caracals reportedly consume the most abundant prey available (Avenant & Nel, 2002). Lastly, lower dietary similarities in areas without range overlap could be influenced in part by distributions of prey species across taxonomic categories used in our analyses, as well as the differences in the habitat preferences of each felid species.

Caracals consumed more diverse prey species compared to jungle cats, in part a consequence of their generalist and opportunistic foraging behavior (Avenant & Nel, 2002). We found that caracals, in general, consumed prey with greater average body mass than jungle cats, likely a consequence of their larger body size. Among co‐occurring carnivores with similar morphology and hunting strategy, co‐occurrence may involve larger predators specializing in larger prey (Rosenzweig, 1966). Co‐occurring pumas (*Pume concolor*) and jaguars (*Panthera onca*) relied mostly on larger mammals than ocelots in the savannas of western Venezuela (Farrell et al., 2000), and larger‐bodied lions consumed larger prey than did the smaller co‐occurring leopards and cheetahs (*Acinonyx jubatus*) in the Kruger National Park, South Africa (Owen‐Smith & Mills, 2007). Our results showed no effect, however, of range overlap on prey average body mass consumed by caracal and jungle cat, with caracal always consuming prey with greater average body mass. The possible underestimation of small prey derived from our data treatment (i.e., conversion to relative frequencies, and use of scat‐only data) may have influenced this result. Lastly, these two species have a limited area of range overlap, and the number of studies reporting caracal and the jungle cat were few, which limited the scope of our comparison.

We found no livestock and little carnivore remains in jungle cat diet, likely because jungle cats are considered small rodent specialists (Rostro‐García et al., 2021). General foraging theory considers a species to be a trophic specialist when it exploits a certain resource regardless of its availability (Glasser, 1982; Malo et al., 2004). Jungle cats have long legs, slender builds, small heads, and tawny pelages which are considered adaptations for preying on small rodents in grasslands (Nowell & Jackson, 1996). Furthermore, jungle cats prey predominantly on rodents exhibiting nocturnal activity (Majumder et al., 2011; Mukherjee et al., 2004; Rostro‐García et al., 2021), suggesting that unlike caracals, jungle cats are more specialized predators and select rodents. Similar specialization has been documented in other felids including snow leopards (*Panthera uncia*), that are dietary specialists of mountain‐dwelling ungulates at higher elevations, even when this prey is much less abundant than livestock (Johansson et al., 2015; Lovari et al., 2013). Alternatively, dietary studies of jungle cats may not have been conducted in areas where vulnerable livestock (i.e., poultry) occurred. Furthermore, that livestock, particularly poultry were not detected in diets could be attributed to use of scats or stomachs for analyses, as it is difficult to determine the bird species and many authors may have grouped all bird species, potentially including poultry.

Studies on diets are important to predict the viability of each species in its habitat (Wang, 2002). Diet analyses of free‐ranging carnivores can improve our understanding of their potential effects on prey populations, ecology, and potential competition with other predators (Jedrzejewski et al., 2002; Wachter et al., 2012). Our results have important implications for caracal and jungle cat ecology and conservation, suggesting that greater prey diversity in areas of range overlap may be a mechanism allowing their co‐occurrence. Additionally, it appears that the larger size and opportunistic feeding behavior of caracals allows consumption of more diverse prey species compared to jungle cats, which in turn may facilitate co‐occurrence between these two felid species in areas of range overlap.

## AUTHOR CONTRIBUTIONS

**Jamshid Parchizadeh:** Conceptualization (equal); data curation (equal); investigation (equal); methodology (equal); supervision (supporting); writing – original draft (lead); writing – review and editing (lead). **Sarah L. Schooler:** Conceptualization (equal); data curation (equal); formal analysis (equal); methodology (equal); software (equal); writing – review and editing (supporting). **Sahar Rezaei:** Writing – original draft (supporting). **Mohammad Ali Adibi:** Data curation (equal); writing – review and editing (equal). **Jerrold L. Belant:** Conceptualization (equal); funding acquisition (lead); methodology (equal); project administration (equal); supervision (lead); writing – review and editing (equal). **Mariano G. Arias:** Data curation (equal); formal analysis (equal); methodology (equal); software (equal).

## CONFLICT OF INTEREST STATEMENT

The authors declare that they have no known competing financial interests or personal relationships that could have appeared to influence the work reported in this paper.

## Supporting information


Appendix S1

Appendix S2

Appendix S3

Appendix S4

Appendix S5

Table S1

## Data Availability

Authors agree to deposit data supporting their accepted paper in Dryad data repository. Doi:10.5061/dryad.z8w9ghxhw.
