# Supplementary material for: A review of caracal and jungle cat diets across their geographical ranges during 1842–2021
Source: Ecol Evol. 2023 May 25;13(5):e10130. doi: 10.1002/ece3.10130 (PMC10212689; doi:10.1002/ece3.10130)
Supplement: Supplementary file 5 — Appendix S5 [file ECE3-13-e10130-s003.docx]

**References listed in Appendix 1**

Adhya, T. 2014. Habitat use and diet of two sympatric felids - the Fishing cat (Prionailurus viverrinus) and the Jungle cat (Felis chaus) - in a human-dominated landscape in suburban Kolkata. Tata Institute of Fundamental Research, Mumbai.

Adibi, M. A., M. Karami, and M. Kaboli. 2014. Study of seasonal changes in habitat suitability of *Caracal caracal schmitzi* (Maschie 1812) in the central desert of Iran. 5:96–106.

Allayarov, A. M. 1964. Materialy po ekologii I geografincheskomu rasprostranenie kamyshovovo kota v Uzbekistane (Material on the ecology and geographic distribution of the jungle cat in Uzbekistan). Uzbekskii Biologicheskii Zhurnal 2:46–50.

Avenant, N. L. 1993. The caracal, Felis caracal caracal Schreber, 1776, as predator in the West Coast National Park. University of Stellenbosch MSc:213.

Avenant, N. L., and J. A. J. Nel. 1997. Prey use by four syntopic carnivores in a strandveld ecosystem. African Journal of Wildlife Research 27:86–93.

Avenant, N. L., and J. A. J. Nel. 2002. Among habitat variation in prey availability and use by caracal Felis caracal. Mammalian Biology 67:18–33.

Azzaroli, L., and A. M. Simonetta. 1966. Carnivori della somalia ex italiana. Monitore Zoologico Italiano, Supplemento 1:102–195.

Bekker, S. J. 1994. Die invloed van sosio-ekologiese veranderlikes op die aard en omvang van die probleemdierkwessie in Suid-Oos Kaapland [The influence of socio-ecological variables on the nature and extent of the problem animal issue in the Southeastern Cape]. Stellensbosch University, Stellenbosch, South Africa. <https://scholar.sun.ac.za/bitstream/handle/10019.1/58356/bekker_invloed_1994.pdf>.

Bester, J. 1982. Die gedragsekologie en bestuur van die silwervos Vilpes chama (A. Smith) met spesiale verwysing na die Oranje-vrystaat. Universiteit van Pretoria, Pretoria, South Africa.

Bothma, J. du P. 1965. Random observations of the food habits of certain Carnivora of South Africa. Fauna and Flora 16.

Braczkowski, A., L. Watson, D. Coulson, J. Lucas, B. Peiser, and M. Rossi. 2012. The diet of caracal, Caracal caracal, in two areas of the southern Cape, South Africa as determined by scat analysis. African Journal of Wildlife Research 42:111–116.

Chernyshev, V. I. 1948. K biologii kamyshovovo kota v Tadzhikistane [Biology of the jungle cat in Tadzhikistan]. Soobshcheniya Tadzhikskogo Filiala Akademii Nauk SSR 5:33–35.

Dobamo, T. 2019. Ecology of Caracals and their distribution in Africa: a review paper. Journal of Biology, Agriculture, and Healthcare 9:51–63. <www.iiste.org>.

Dominis, J., and M. A. Edey. 1968. The Caracal. Pages 109–119 *in*. Cats of Africa. 1st Editio. Time-life Books.

Dragesco-Joffé, A. 1993. La vie sauvage au Sahara [Wildlife in the Sahara]. Delachaux et Niestlé, Paris, France.

Drouilly, M., N. Nattrass, and M. J. O’Riain. 2018. Dietary niche relationships among predators on farmland and a protected area. Journal of Wildlife Management 82:507–518.

Drouilly, M., N. Nattrass, and M. J. O’Riain. 2020. Global positioning system location clusters vs. scats: comparing dietary estimates to determine mesopredator diet in a conflict framework. Journal of Zoology 310:83–94.

Farhadinia, M. S., H. Akbari, M. Beheshti, and A. Sadeghi. 2007. Ecology and status of the Caracal, *Caracal caracal*, in the Abbasabad Naein Reserve, Iran. Zoology in the Middle East 41:5–10.

Forester, D., and T. Tear. 1988. Caracal cunning. PDO News 8–11.

Ghoddousi, A., T. Ghadirian, and H. Fahimi. 2009. Status of caracal in Bahram’gur Protected Area , Iran. Cat News 50.

Grinberg, V. B. 1933. Rys’ i rosomakha, ikh promysly i znachenie V okhotnich’em khozyaistve SSSR [Lynx and wolverine, their exploitation and importance in the hunting economy of the USSR]. Moscow-Leningrad.

Grobler, J. H. 1981. Feeding behaviour of the caracal Felis caracal Schreber 1776 in the Mountain Zebra National Park. South African Journal of Zoology 16:259–262.

Grzimek, H. C. 1975. The Caracal. Page 312 *in*. Grzimek’s Animal Life Encyclopedia. Van Nogrand Reilhold Company, New York, NY.

Harrison, D. L., and P. J. J. Bates. 1991. Felidae. Pages 156–172 *in*. The Mammals of Arabia. 2nd edition. Harrison Zoological Museum, Sevenoaks, UK.

Van Heezik, Y. M., and P. J. Seddon. 1998. Range size and habitat use of an adult male caracal in northern Saudi Arabia. Journal of Arid Environments 40:109–112.

Heptner, V. G., and A. N. Formozov. 1941. Mlekopitayuschie Dagestana [Mammals of Dagestan]. Sbornik Trudov Zoologicheskogo Muzeya Moskovskogo Universiteta 6.

Heptner, V. G., and A. A. Sludskii. 1992. Mammals of the Solviet Union Volume II Part 2 (P.M. Rao, Trans.). V. G. Heptner, N. P. Naumov, R. S. Hoffmann, and V. S. Kotehkar, editors. Smithsonian Institution Libraries. (Original work published 1972), Washington, D.C.

Ishunin, G. I. 1965. K biologii kamyshovovo kota v yuzhnom Uzbekistane [Biology of the jungle cat in southern Uzbekistan]. Zoologicheskii Zhurnal 4:630–632.

Jansen, C., A. J. Leslie, B. Cristescu, K. J. Teichman, and Q. Martins. 2019. Determining the diet of an African mesocarnivore, the caracal: scat or GPS cluster analysis? Wildlife Biology 2019:1–8.

Jerdon, T. C. 1874. The mammals of India : a natural history of all the animals known to inhabit continental India. John Wheldon, London, UK.

Johnsingh, A. J. T. 1983. Large mammalian prey-predators in Bandipur. Journal of the Bombay Natural History Society 80:1–57.

Khalaf-von Jaffa, N. A. 2006. Felidae Palaestina: the wild cats of Palestine. Gazelle: The Palestinian Biological Bulletin.

Khan, A. A., and M. A. Beg. 1986. Food of some mammalian predators in the cultivated areas of Punjab. Pakistan J.Zool. 18:71–79.

Kingdon, J. 1977. Caracal (Felis caracal). Pages 330–339 *in*. East African Mammals: An Atlas of Evolution in Africa, Volume 3, Part A. 1st Editio. Academic Press, London.

de la Fuente, F. 1970. World of wildlife: hunters and hunted of the savannah. Orbis Publishing Ltd, London, UK.

Lay, D. M. 1967. A Study of the Mammals of Iran of 1962-63. E. G. Nash, P. M. Williams, and J. C. Moore, editors. Fieldiana: Zoology Volume 54. Volume 54. Field Museum of Natural History, Chicago, Illinois.

Leighton, G. R. M., Bishop, J. M., O’Riain, M. J., [Broadfield](https://link.springer.com/article/10.1007/s11252-020-00946-y#auth-Joleen-Broadfield), J., [Meröndun](https://link.springer.com/article/10.1007/s11252-020-00946-y#auth-Justin-Mer_ndun), J., [Avery](https://link.springer.com/article/10.1007/s11252-020-00946-y#auth-Graham-Avery), G., [Avery](https://link.springer.com/article/10.1007/s11252-020-00946-y#auth-D__Margaret-Avery), D. M. and [Serieys](https://link.springer.com/article/10.1007/s11252-020-00946-y#auth-Laurel_E__K_-Serieys), L. E. K. 2020. An integrated dietary assessment increases feeding event detection in an urban carnivore. Urban Ecosystems. 23:569–583.

Majumder, A., K. Sankar, Q. Qureshi, and S. Basu. 2011. Food habits and temporal activity patterns of the Golden Jackal *Canis aureus* and the Jungle Cat *Felis chaus* in Pench Tiger Reserve, Madhya Pradesh. Journal of Threatened Taxa 3:2221–2225.

Melville, H. I. A. S., J. du P. Bothma, and M. G. L. Mills. 2004. Prey selection by caracal in the Kgalagadi Transfrontier Park. African Journal of Wildlife Research 34:67–75.

Mendelssohn, H. 1989. Felids in Israel. Cat News 10:2–4.

Mills, M. G. L. 1978. Foraging Behaviour of the Brown Hyaena. Zeitschrift für Tierpsychologie 48:113–141.

Momeni, S., M. Malekian, and M. R. Hemami. 2019. Molecular versus morphological approaches to diet analysis of the caracal (Caracal caracal). Mammalia 83:586–592.

Moolman, L. C. 1984. ’n vergelyking van die voedingsegwoontes van die rooikat Felis caracal binne en buite die Bergkwagga Nasionale Park. Koedoe 27:121–129.

Moolman, L. C. 1986. Aspekte van die ekologie en gedrag van die rookat *Felis caracal* Schreber, 1776 in die Bergkwagga Nasionale Park en op die omliggende plase. University of Pretoria, Pretoria, South Africa.

Mukherjee, S. 1989. Ecological separation of four sympatric carnivores in Keoladeo Ghana National Park, Bharatpur, Rajasthan, India. Saurashtra University, Rajkot.

Mukherjee, S., S. P. Goyal, A. J. T. Johnsingh, and M. R. P. L. Pitman. 2004. The importance of rodents in the diet of jungle cat (*Felis chaus*), caracal (*Caracal caracal*) and golden jackal (*Canis aureus*) in Sariska Tiger Reserve, Rajasthan, India. Journal of Zoology 262:405–411.

Neils, A. M. 2018. Caracals in a heterogenous landscape: resolutions for human-carnivore conflicts. University of Arizona.

Niethammer, J. 1966. Zur ernährung des sumpluchses (Felis chaus Güldenstaedt, 1776) in Afghanistan. Zeitschrift für Säugetierkunde 31:393–394.

Novikov, G. A. 1962. Carnivorous mammals of the fauna of the USSR. Israel Program for Scientific Translations, Jerusalem, Israel.

Nowell, K., and P. Jackson. 1996. Wild Cats: Status Survey and Conservation Action Plan. International Union for Conservation of Nature and Natural Resources, Gland, Switzerland.

Ognev, S. I. 1935. Zveri SSSR i prilezhaschikh stran [Animals of the USSR and adjacent countries]. Volume 3. Moscow-Leningrad.

Palmer, R., and N. Fairall. 1988. Caracal and African wild cat diet in the Karoo National Park and the implications thereof for hyrax. South African Journal of Wildlife Research 18:30–34.

Pienaar, U. D. V. 1964. The small mammals of the Kruger National Park - A systematic list and Zoogeography. Koedoe. Volume 7.

Pienaar, U. D. V. 1969. Predator-prey relationships amongst the larger mammals of the Kruger National Park. Koedoe 108–156.

Pohl, C. F. 2015. The diet of caracal (Caracal caracal) in the Southern Free State. University of the Free State.

Prakesh, I. 1959. Food of some Indian desert mammals. Journal of Biological Sciences 2:100–109.

Pringle, J. A., and V. L. Pringle. 1979. Observations on the Lynx *Felis caracal* in the Bedford District. South African Journal of Zoology 14:1–4.

Rathore, F. S., and V. Thapar. 1984. Behavioral observations of leopard and jungle cat in Ranthambhor National Park and Tiger Reserve, Rajasthan. Pages 136–139 *in*. The plight of the cats: proc. meeting and workshop of the IUCN/SSC cat specialist group at Kanha National Park, Madhya Pradesh, India 9-12 April. IUCN/SSC Cat Specialist Group, Bougy-Villars, Switzerland.

Rautenbach, I. L. 1978. The mammals of the Transvaal. University of Natal, Pietermaritzburg, South Africa.

Roberts, T. J. 1977. The mammals of Pakistan. First. Ernest Benn Limited, London.

Rowe-rowe, D. T. 1978. The small carnivores of Natal. The Lammergeyer 3:38–39.

Sapozhenkov, Y. F. 1960. Karakal pod Repetekom [Caracal around Repetek]. Priroda 2:107–108.

Sapozhenkov, Y. F. 1962. Ob ekologii karakala (*Felis caracal* Mull.) v Karakumakh [On the ecology of the caracal (*Felis caracal* Mull.) in the Karakum. Zoologicheskii Zhurnal 41:1111–1112.

Schaller, G. B. 1967. The deer and the tiger: a study of wildlife in India. University of Chicago Press, Chicago, Illinois.

Shortridge, C. G. C. 1934. The Caracal. Pages 97–100 *in*. The Mammals of South West Africa. Volume 1. William Heinemann Ltd, London, UK.

Skinner, J. D., and C. Chimimba. 2005. Caracal caracal. Pages 397–401 *in*. The Mammals of the Southern African Sub-region. 3rd Editio. Cambridge University Press, Cambridge, UK.

de Smet, K. J. M. 1989. Distribution and habitat choice of larger mammals in Algeria, with special reference to nature protection. Ghent State University, Ghent, Belgium.

Smithers, R. H. N. 1971. *Felis caracal*. Pages 119–122 *in*. The mammals of Botswana. Museum Mem. Trustees of the National Museums of Rhodesia, Salisbury, South Africa (Rhodesia).

Smithers, R. H. N., and V. J. Wilson. 1979. Check list and atlas of the mammals of Zimbabwe Rhodesia. The Trustees of the National Museums and Monuments, Salisbury, Zimbabwe Rhodesia.

Stewart, D. R. . 1963. The Arabian oryx (Oryx leucoryx Pallas). African Journal of Ecology 1:103–117.

Stuart, C., and M. Stuart. 2007. Diet of leopard and caracal in the northern United Arab Emirates and adjoining Oman territory. Cat News 46:30–31.

Stuart, C. T. 1981. Notes on the mammalian carnivores of the Cape Province, South Africa. Bontebok 1:1–58.

Stuart, C. T. 1982. Aspects of the biology of the Caracal (Felis caracal Schreber 1776) in the Cape Province, South Africa. 204 pp.

Stuart, C. T., and G. C. Hickman. 1991. Prey of caracal *Felis caracal* in two areas of Cape Province, South Africa. Journal of African Zoology 105:373–381.

Tehsin, R., and F. Tehsin. 1990. Jungle cat Felis chaus and grey junglefowl Gallus sonneratii. Journal of the Bombay Natural History Society 87:144.

Vereschagin, N. K. 1942. Katalog zverei Azerbaidzhana (Catalog of Azerbaidzhan animals). Izd-vo Akademii nauk Azerbaĭdzhanskoĭ SSR, Baku, Azerbaijan.

Vigne, G. T. 1842. Travels in Kashmir, Ladak, Iskardo, the countries adjoining the mountain-course of the Indus and the Himalaya, north of the Punjab with map, Volume 1. 1st edition. Henry Colburn, London, UK.

Viljoen, S., and D. H. S. Davis. 1973. Notes on stomach contents analyses of various carnivores in Southern Africa (Mammalia: Carnivora). Annals of the Transvaal Museum 28:354–363.

Weisbein, Y. 1989. The biology and ecology of the caracal Felis caracal in the Aravah Valley of Israel. Cat News 12:20–22.

Wilson, B. 2015. The black-footed cat Felis nigripes (Burchell, 1824): a review of the geographical distribution and conservation status. Tshwane University of Technology, Pretoria, South Africa.

Wilson, V. J. 1966. Predators of common duiker, Sylvicapra grimmea in eastern Zambia. Arnoldia (Rhodesia) 2:1–7.
